# Scurvy in a Pediatric Patient: A Rare Diagnosis in the Modern World

**DOI:** 10.7759/cureus.82766

**Published:** 2025-04-22

**Authors:** Evdokia Pavlidou, Athanasios Georgoulis, Sofia Zarenti, Aikaterini Elisavet Doufexi

**Affiliations:** 1 School of Dentistry, Aristotle University of Thessaloniki, Thessaloniki, GRC; 2 Medical School, Aristotle University of Thessaloniki, Thessaloniki, GRC; 3 Preventive Dentistry, Periodontology and Implant Biology, Aristotle University of Thessaloniki, Thessaloniki, GRC

**Keywords:** ascorbic acid, gingival hemorrhage, nutrition disorders, scurvy, vitamins

## Abstract

Scurvy is the oldest-known nutritional disorder caused by a sustained lack of ascorbic acid (vitamin C). Despite its rarity in developed countries, scurvy has been increasingly reported in recent years in pediatric patients, particularly those with selective or restricted feeding. It mainly affects the musculoskeletal system, the skin, and the oral cavity, causing gingival bleeding. Diagnosing scurvy can be difficult for clinicians because of its nonspecific symptoms, often leading to extensive testing and delayed identification of the condition. The treatment is based on changing eating habits and taking supplements with vitamin C. This study aims to present a case report of a 12-year-old child with oral manifestations of scurvy, discussing the differential diagnosis, clinical findings, and therapeutic approach, while also reviewing current literature on the topic.

## Introduction

Scurvy is a disease resulting from prolonged vitamin C deficiency, leading to impaired collagen synthesis and widespread connective tissue dysfunction. Historically, scurvy was prevalent among sailors and individuals with limited access to fresh fruits and vegetables [1]. However, recent reports indicate an increasing number of cases in developed countries, particularly among children with restrictive diets, autism spectrum disorders, or neurological impairments [2,3].

Vitamin C plays a critical role in maintaining the integrity of blood vessels, promoting wound healing, and supporting immune function. Its deficiency can lead to various systemic and oral manifestations, including fatigue, musculoskeletal pain, anemia, gingival inflammation, and spontaneous bleeding [1-3]. Since this disease is rare and the symptoms are not well defined, it is misdiagnosed as a hematological, autoimmune, or infectious disease, which results in failure of appropriate management [4]. This paper aims to present a case of pediatric scurvy, including the clinical features, diagnosis, management, and the importance of early recognition of oral signs.

## Case presentation

**Figure 1:**
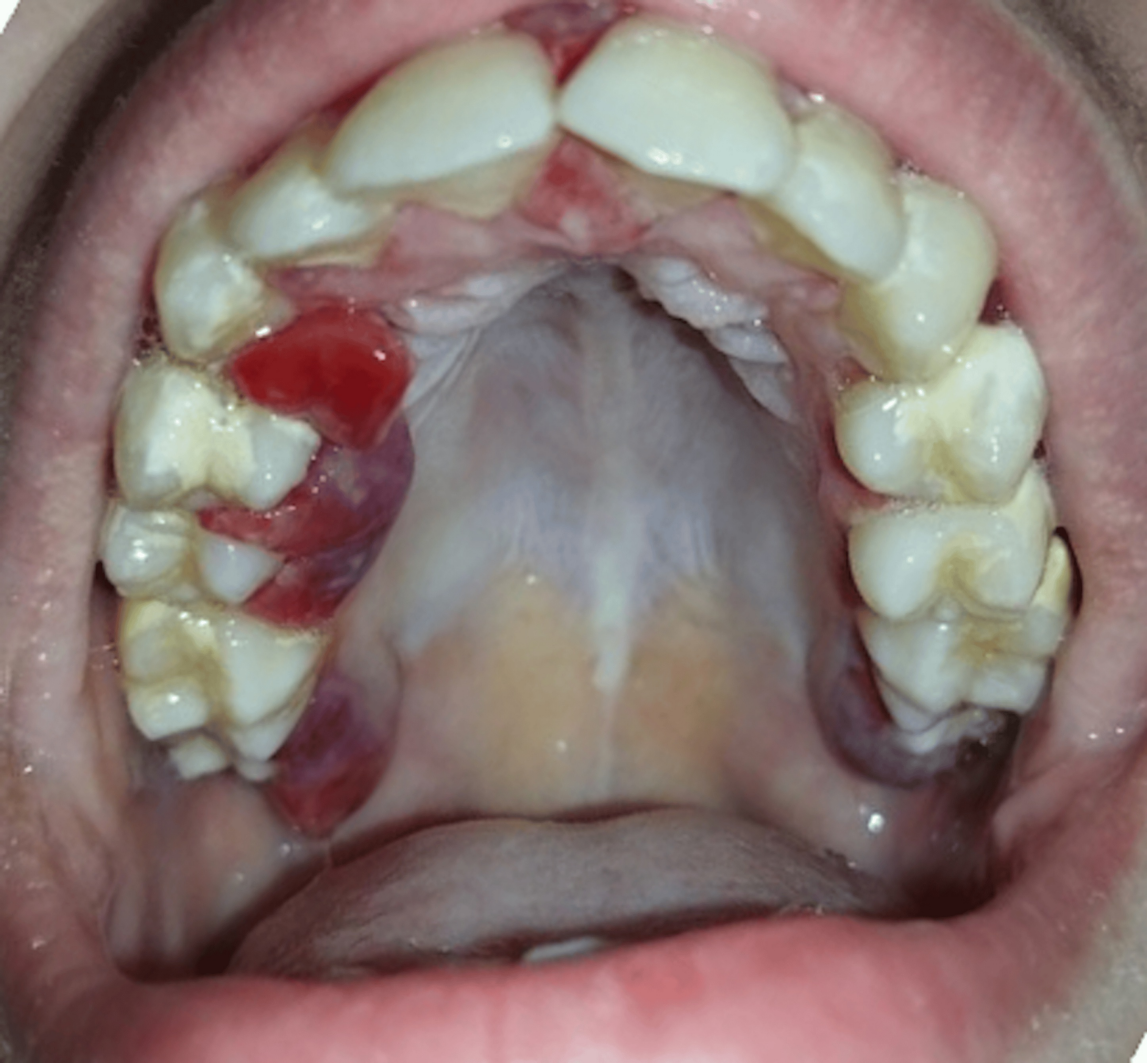
Clinical image of the palatal view The image shows erythematous and swollen gingiva.

**Figure 2:**
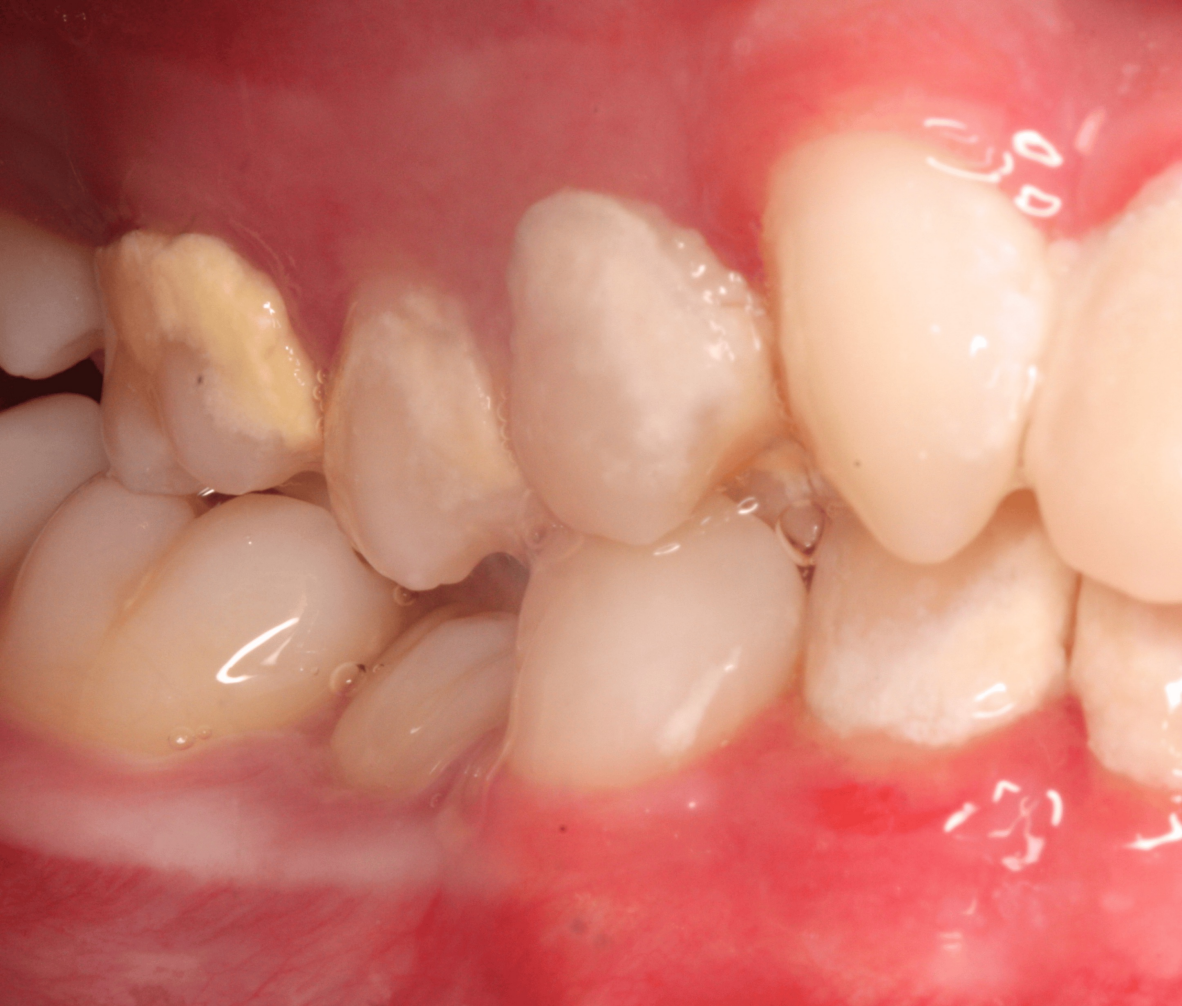
Clinical photograph showing gingivitis and calculus

A 12-year-old boy with no significant medical history presented with pale-yellow skin, particularly noted around the face, without any purpuric or petechial lesions visible, fatigue, and oral lesions. He reported gingival bleeding during tooth brushing, along with weakness and malaise. No signs of petechiae, ecchymosis, or hyperkeratosis were noted upon dermatological examination. His weight was 30 kg, height 140 cm, and BMI 15.3 kg/m² (underweight). Clinical examination revealed generalized gingival inflammation with swollen and hemorrhagic gingivae with considerable plaque accumulation and a high plaque index, suggesting poor oral hygiene as a compounding factor in the gingival presentation (Figures 1, 2). The buccal mucosa, lips, and tongue remained unaffected. There was no evidence of ulceration, tumors, or palpable abnormalities in the jaws, submandibular triangle, or temporomandibular region joint.

**Figure 3:**
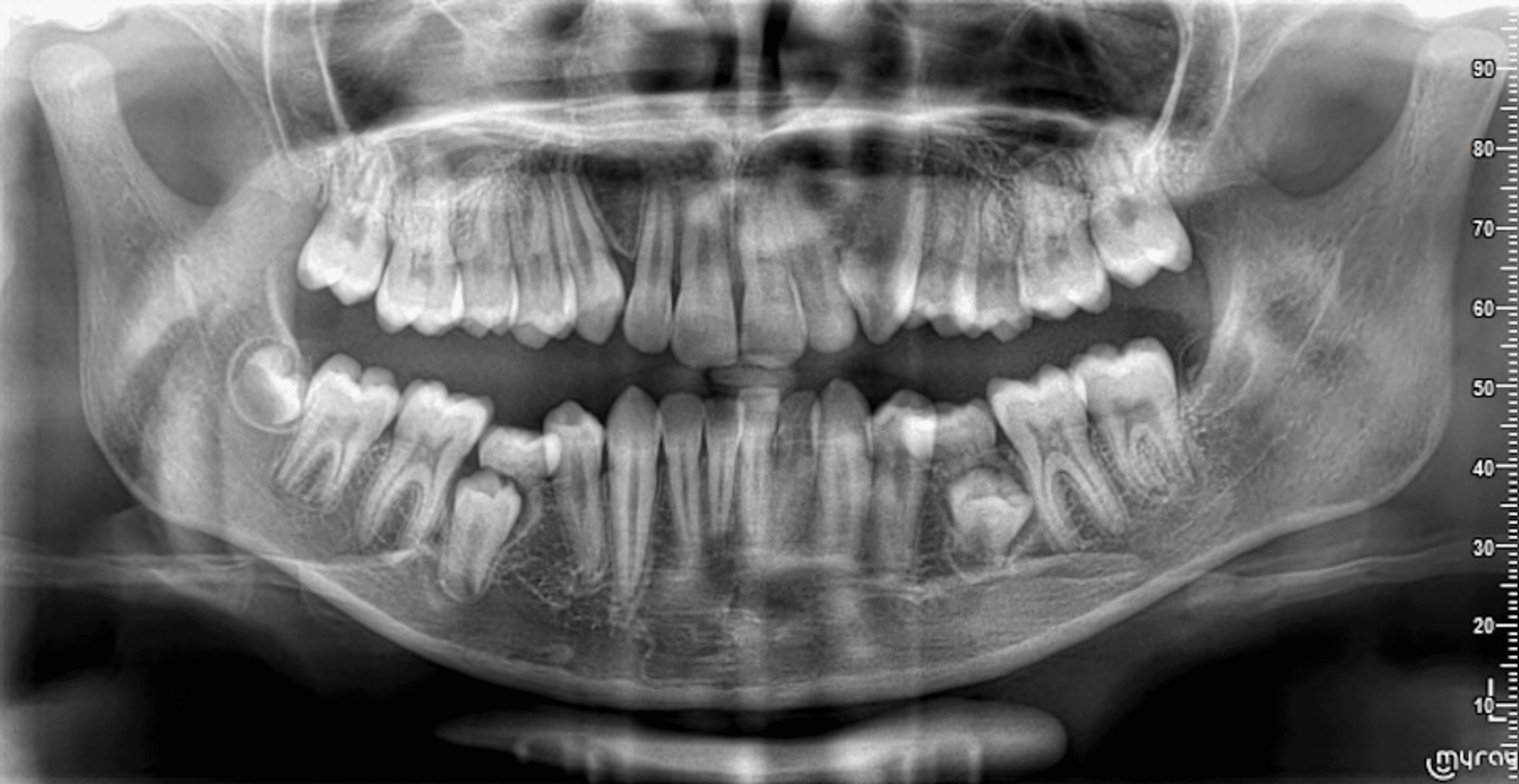
Normal panoramic radiographic appearance

The gingival enlargement, though resembling a tumorous appearance, raised differential concerns, including reactive, inflammatory, or neoplastic conditions, as many diseases exhibit similar oral symptoms. The differential diagnosis considered a broad spectrum of conditions that can present with similar oral findings. These included infectious diseases (e.g., necrotizing ulcerative gingivitis, herpetic stomatitis), autoimmune disorders (e.g., pemphigus vulgaris, mucous membrane pemphigoid, systemic lupus erythematosus), and hematologic malignancies such as acute leukemia. Moreover, other conditions are malignancy, such as acute leukemia and lymphoma, and bacterial infections like syphilis, tuberculosis, or actinomycosis, which may involve oral ulceration, swelling, or gingival changes. No abnormalities were noted on the orthopantomogram (OPG) (Figure 3). Biochemical blood tests and biopsy were conducted to reach a diagnosis. 

Laboratory tests revealed folate deficiency, low ferritin levels, and a hematocrit of 25. Elevated erythrocyte sedimentation rate (ESR) and C-reactive protein (CRP) suggested significant inflammation. The neutrophil-to-lymphocyte ratio was inverted. Critically, plasma vitamin C levels were <0.2 mg/dL, confirming scurvy. Unexpectedly, the inversion of the neutrophil-to-lymphocyte ratio (NLR) further complicated the differential diagnosis (Table 1).

**Table 1:** Blood test results CRP: C-reactive protein, ESR: Erythrocyte sedimentation rate, RBC: Red blood cell, Hgb: Hemoglobin, HCT: Hematocrit, MCV: Mean corpuscular volume, RDW: Red cell distribution width, PLT: Platelet count.

	Results	Normal Values
Ferritin	36.41 ng/ml	7.00-140.00
CRP	26.3 mg/l	<10
Iron	81.7 mgr/dl	53-120
ESR	35	10-15
RBCs	3.82	3.9-5.5
Hgb	7.8 gr/dl	12-16
HCT	25.3 %	36-47
MCV	66.2 fl	79-98
RDW	18.7 %	11.5-14.9
PLT	259 x10³/μL	150-450

Histological examination showed follicular hyperkeratosis, hemorrhagic signs, including perivascular bleeding and extravasation of red blood cells in the dermis, hyperkeratosis, and hematologic abnormalities, such as microcytic anemia with low hemoglobin, low hematocrit, and elevated RDW. The key histopathological feature contributing to the diagnosis was the irregular organization of collagen within the endothelial cell matrix. Upon dietary history evaluation, the patient was found to consume a highly restrictive diet consisting mainly of cookies, waffles, and spaghetti, reinforcing the diagnosis of scurvy.

**Figure 4:**
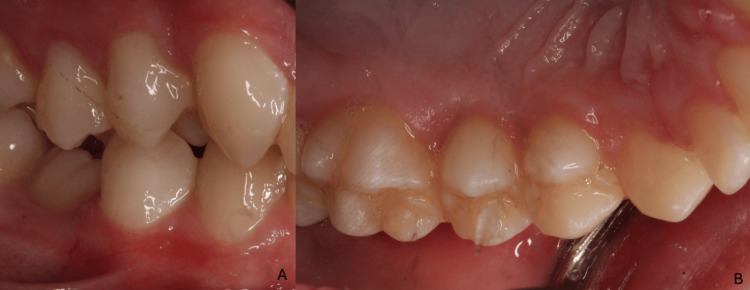
Clinical photographs following the treatment The treatment included debridement, oral hygiene maintenance, a balanced diet, and vitamin C supplementation.

The treatment plan was straightforward and involved the administration of vitamin C supplementation alongside a diet abundant in fruits and vegetables. Notably, spontaneous bleeding, as well as both oral and general symptoms, showed significant improvement within a few days. Additionally, intravenous iron was administered to elevate serum iron levels, resulting in a rapid resolution of symptoms. One-month post-treatment, follow-up tests were conducted, which indicated normal values for ascorbic acid, hematocrit, iron, CRP, and tumor markers. A follow-up appointment three months later at the dental office showed no signs of recurrence, as evidenced by the reduction in gingival swelling and hemorrhage (Figures 4A, 4B). The patient was enrolled in a follow-up program with both the pediatrician and dietitian to better support healthy eating habits.

## Discussion

Vitamin C

Vitamin C, also known as ascorbic acid, is a water-soluble vitamin and the enolic form of alpha-keto lactone [5]. It plays a vital role in immune function, bone and dental health, and tissue healing [5,6]. It is primarily absorbed in the distal small intestine and stored in organs such as the pituitary gland, adrenal glands, brain, and white blood cells [5]. The human body cannot synthesize vitamin C; therefore, it must be obtained from dietary sources such as citrus fruits and vegetables [5,7]. The recommended daily allowance (RDA) of vitamin C is 15-45 mg for children (1-13 years) to 65-75 mg for adolescents (14-18 years) [8]. Certain groups, including pregnant individuals and older adults, require up to 120 mg per day [5]. Vitamin C contributes to collagen hydroxylation, which is essential for maintaining blood vessel integrity. A deficiency leads to weakened, fragile vessels, increasing the risk of bleeding and impaired wound healing [5,9].

Clinical features 

Initial signs of significant vitamin C deficiency include fatigue, reduced exercise tolerance, feelings of depression, irritability, and a mild fever [8,10]. Oral manifestations occur early and include gingival swelling, increased fragility, and spontaneous bleeding with minor pressure [8,11]. Pediatric patients with scurvy may present with musculoskeletal symptoms, including bone pain, muscle soreness, skeletal muscle degeneration, and arthritis [8,12,13]. Although scurvy may present with antalgic gait and musculoskeletal weakness in some pediatric patients, these were not observed in this case [14]. In addition, mucocutaneous symptoms may manifest as petechiae, ecchymoses, edema, hyperkeratosis, bruising, and alopecia [8,10,13]. While impaired wound healing or minor injuries are recognized features of scurvy, in this case, the patient did not exhibit any post-traumatic or surgical delays in tissue repair [8,10,12].

Proptosis resulting from orbital hemorrhage can serve as a manifestation of scurvy [8]. Conjunctival dryness is also noted as a significant symptom [10]. Additionally, research indicates that neuropsychiatric symptoms, such as irritability and sleep disturbances, have been reported [14]. Other rare but recognized clinical manifestations of scurvy include pulmonary hypertension, cardiac hypertrophy, and compromised adrenal and bone marrow function [8].

Differential diagnosis

Scurvy exhibits clinical similarities with a range of conditions, including necrotizing ulcerative gingivitis, acute herpetic gingivostomatitis, leukemia, desquamative gingivitis associated with autoimmune disorders, bleeding disorders, and gingival hyperplasia resulting from the use of antiepileptic medications [4,8]. When a patient presents with ecchymoses, petechiae, and mucosal bleeding, it is essential to consider various potential underlying causes, including vascular abnormalities, platelet disorders, and coagulopathies [10,15]. The presence of symptoms such as limb pain, particularly nocturnal pain, cachexia, pallor, petechiae, ecchymosis, and gingival hypertrophy necessitates a thorough evaluation for possible malignancy [2,15]. Musculoskeletal symptoms such as limb swelling, fever, and elevated inflammatory markers may suggest osteoarticular infections, osteomyelitis, septic arthritis, myositis, or spondylodiscitis [2,16].

Diagnosis

Healthcare providers should diligently inquire about dietary habits and take into account the possibility of nutritional deficiencies; otherwise, the diagnosis of scurvy can easily be overlooked [13]. Dentists play a crucial role in identifying nutritional deficiencies in children, especially those with autism, learning difficulties, or selective eating habits, as oral symptoms often appear early [4,11]. The primary diagnostic approach includes baseline blood tests [4,6]. Vitamin C deficiency is an important diagnostic factor in the case of scar swelling [4,6,10,16]. A low plasma level of vitamin C (plasma ascorbate concentration of <0.2 mg/dl) is a specific indicator of scurvy [5,8]. Given that multiple micronutrient deficiencies can coexist, clinicians should evaluate levels of zinc, iron, folate, and vitamin B12 [8,13].

Radiographs are essential tools that greatly assist in the diagnostic process. Classical signs of scurvy on radiographic imaging include the widespread osteopenia with a pencil-thin cortex, the Pelkan spur (metaphyseal spurs), the Trummerfeld zone (a lucent metaphyseal band known as the 'scorbutic zone'), the Fränkel lines (calcification at the metaphysis), and the Wimberger ring sign (epiphyseal calcifications) [7,8,12,13,16,17].

## Conclusions

While scurvy is uncommon in today's developed nations, it is still a significant medical issue, especially among children who have limited diets. Dentists are vital in identifying vitamin C deficiency at an early stage since oral signs frequently emerge before the onset of systemic symptoms. A comprehensive dietary history and specific laboratory tests are crucial for a correct diagnosis, avoiding unwarranted medical procedures, and facilitating timely treatment. This situation emphasizes the necessity of being aware of nutrition in children's health and illustrates the demand for more education regarding the dangers of dietary deficiencies. Timely identification and intervention can avert complications and promote swift recovery, highlighting the importance of a nutritious diet that is abundant in essential vitamins.
